# Impact of China’s digital economy development on the health of middle-aged and older people: an air pollution-based perspective

**DOI:** 10.3389/fpubh.2023.1281405

**Published:** 2023-12-21

**Authors:** Jing Wu, Qing Li

**Affiliations:** ^1^School of Economics and Management, Xinjiang University, Wulumuqi, China; ^2^Center for Innovation Management Research of Xinjiang, Wulumuqi, China

**Keywords:** digital economy, air pollution, health, China, middle and old age

## Abstract

China has shown good momentum on the road of digital economy development, however, it is also rapidly entering an aging society. Exploring the health effects of the digital economy is of positive significance for realizing healthy aging in China. This paper focuses on the relationship between the digital economy and the health of middle-aged and older people using microdata from the China Health and Retirement Longitudinal Study (CHARLS) 2011–2018 and macrodata from Chinese cities. The study found that the digital economy showed a significant inverted U-shaped relationship on the health of middle-aged and older people. The results of subgroup regressions indicated heterogeneity in this effect across gender, education level, urban/rural and region. Individual health in female, highly educated, and urban groups is more closely related to the digital economy. Middle-aged and old groups in the western region are better able to enjoy the dividends of the digital economy, while middle-aged and old groups in the eastern region are more negatively affected by the digital economy. In the lead-up to the development of the digital economy, individual health can be promoted by narrowing the urban–rural income gap and increasing basic medical resources, while in the later stage of the development of the digital economy, it manifests itself in inhibiting the level of individual health by widening the urban–rural income gap and lowering the level of basic medical resources. In addition, air pollution exhibits a positive moderating effect between the digital economy and individual health, suggesting that air pollution reinforces the impact of the digital economy on health. Expansive analyses indicate that the digital economy has a negative impact on physiological health.

## 1 Introduction

The rise and advancement of the digital economy, a new type of economy, is a result of the fusion between modern information technology and the development of the world economy and the way of human production and life. The digital economy, led by the Internet, big data and other digital technologies, is developing rapidly and is changing production and consumption patterns globally (1). At the beginning of the development of the Internet in the last century, Don Tapscott of the United States used the term digital economy in 1996, and he is considered to be one of the first to propose the concept of the “digital economy” (2). He regards the digital economy as an economic system that is developed by ICT. The term digital economy took shape in 1998 when the United States Department of the Treasury issued the report The Emerging Digital Economy. The dynamic development of the digital economy has resulted in no uniformity in its meaning. Since its introduction, the digital economy has been constantly enriched and perfected in terms of its content and scope. A representative viewpoint acknowledges that the digital economy can be regarded as an economic activity that encompasses digital information as a crucial element, employs the Internet as a medium, driven by information and communications technology, with the potential to optimize economic structure and promote economic development (3).

**Figure 1:**
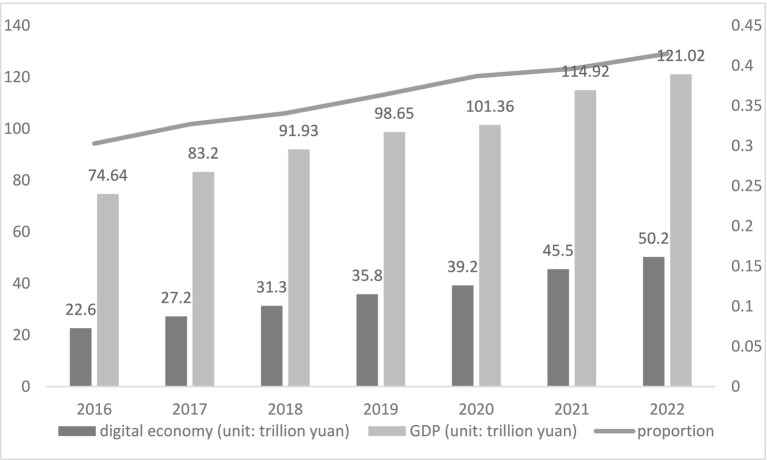
Scale of China’s digital economy and its share of GDP.

China provides a good example in the rapid development of the digital economy. Figure 1 illustrate the development of China’s digital economy. The Digital China Development Report (2022) released by China’s National Internet Information Office (NIIO) shows that China’s digital economy will reach RMB 50.2 trillion in 2022, raising its share of gross domestic product (GDP) to 41.5%, making the total volume firmly ranked second in the world. From 2016 to 2022, China’s digital economy will grow at $4.1 trillion, a compound annual growth rate of 14.2 percent. The rise of the digital economy has sparked the emergence of relevant research, but more studies have not directly proved the relationship between the digital economy and health. Relevant is the relationship between economic growth and health, and there are both positive and negative findings on the impact of the economy on health (4–6). There are also the health effects of information technology, the Internet, and so on. Although many scholars have focused on the impact of the Internet on health, the concept of the digital economy has a significantly broader meaning than it. As mentioned earlier, the Internet is an emerging technology, while the digital economy is an economic system that uses the Internet as a vehicle. Therefore the digital economy and the Internet are fundamentally different. In this context, the health effects and influence mechanism of the digital economy as a new driver of economic growth are the focus of this paper.

The health risks associated with environmental degradation are unquestionable, as the environment is an important factor affecting health. The probability of exposure to pollution exacerbates the health risks posed by the environment (7). Air pollution is one of the greatest environmental threats to human health. Air pollution and health have always been a topic of great concern, and a large number of studies have verified the relationship between the two (8–11). The health risks posed by air pollution can be equated to those of an unhealthy diet and smoking, among others (12). However, research on environmental health in the environmental sciences lacks consideration of economic and social factors, which leads to a lack of relevance and effectiveness in the formulation of environmental health policies. Economics, on the other hand, has the advantage of translating endogenous mechanisms into realistic policy implications. From existing research, it is evident that most scholars incorporate environmental factors when discussing the relationship between the economy and health (13). In addition, there have been many studies focusing on the relationship between the digital economy as an economic activity and pollution (14, 15). Unfortunately, few scholars have analyzed the impact of the digital economy on health. As a result, there is a clear lack of research results on the digital economy with respect to health and the environment. Therefore, we include air pollution in our discussion of the health impacts of the digital economy. The perspective of the study is further extended to the dynamic relationship between environment-health-economy in three dimensions. In exploring aspects of health levels in middle-aged and older adults, we seek to provide new theoretical underpinnings from the perspectives of the digital economy and air pollution.

Given the flourishing of China’s digital economy, how does it affect health and what are the mechanisms of action? Indeed, the relationship between the two may exhibit heterogeneity due to variations in individual characteristics and regional factors.? What role does air pollution play in the relationship between the digital economy and health? In order to answer the above questions, this paper uses microdata from the China Health and Retirement Longitudinal Study (CHARLS) 2011–2018 and macrodata from Chinese cities, combined with econometric modeling to provide answers. Our research consists of three main contributions. First, from the perspective of literature, many studies have examined the economic and social effects and environmental effects of the digital economy, but few studies have focused on the health effects of the digital economy, and this paper expands and supplements the existing studies. Second, in terms of content and methodology, the text combines microdata at the household level and macrodata at the city level in China to study the relationship between the digital economy and health and the mechanism of its influence, and pays attention to the moderating effect under air pollution, realizing an effective interface between theory and the facts that characterize China’s new stage of development. Finally, from a policy perspective, the text provides a reference for accelerating the development of the digital economy, coping with the fact that the population is rapidly aging, and promoting the strategy of a healthy China, and provides research support for the decision-making authorities to introduce policies in a targeted manner. The follow-up of this paper is organized as follows: section 2 presents the theoretical and mechanistic analysis and presents the research hypotheses of the article. Section 3 presents the model construction, sample selection and variable selection of the article. Section 4 presents the analysis of empirical results, including benchmark results, endogeneity discussion, robustness test, heterogeneity analysis, mechanism analysis and moderating effect analysis. Section 5 summarizes the findings and provides relevant recommendations.

## 2 Theory and hypothesis

### 2.1 Effects of the digital economy on health

Socioemotional Selectivity Theory (SST) suggests that middle-aged and older people need close social relationships more than younger adults. Individuals narrow their social networks as they enter the middle and older stages of life to the point where they have more time to maintain intimate relationships. As individuals grow older, they tend to selectively limit their social interactions, focusing on maximizing positive emotional experiences while minimizing emotional risks. The result of this behavioral change is an improved quality of life in old age (16), quality of life is simultaneously an important indicator of individual health (17). From the SST point of view, it can be seen that middle-aged and older groups will narrow their social networks, but the digital economy can improve social networking connections. Therefore, the digital economy helps to maintain social relationships and breaking the spatial limitations of social networks, which can strengthen the social interactions of the older, contribute to the re-socialization of the middle-aged and older groups, and conducive to maintain a positive mindset. Some studies have proved that the development of information technology can enable middle-aged and older people to enhance their social interaction and strengthen their ties with the society, which is conducive to maintaining a positive state of mind (18). In addition, Selective Optimization with Compensation Model (SOC) is an important theory for analyzing the application of technology to the health of older adults (19). “Selection” refers to prioritizing relevant activities, choosing the important and discarding the unimportant; “optimization” refers to focusing resources and trying to achieve the desired goals; and “compensation” refers to finding alternatives, assistive technologies and social support (20). The digital economy facilitates the optimization process of middle-aged and older people, thereby compensating for the negative effects of aging and improving health. The development of the digital economy has increased the breadth and depth of information dissemination and facilitated access to health advice, which can enhance the health-related knowledge of middle-aged and older people and facilitate self-prevention and diagnosis. Digital economy and technological advances complement each other, technological solutionism has penetrated the medical and public health fields (21), and “digital health” is the most intuitive embodiment. Digital Health is themed by internet-centric apps and media to improve healthcare programs, commerce, and connectivity (22). Digital technology has been deeply embedded in telemedicine, genomics, and AI consultation. The COVID-19 global pandemic presents a good opportunity for a range of technology-driven healthcare solutions. Many countries have emphasized the development of digital health technologies, which have contributed to the advancement of overall health (21). This includes GPS data, health databases, facial recognition technology, connected surveillance systems and smartphones for disease monitoring (23). However, when the digital economy grows to a certain point, older persons may be marginalized and become “digital refugees.” Digital refugees are people who have fled the digital world and are unable to assimilate into local cultures (24). The application and value realization of the digital economy requires individuals to have certain learning ability, knowledge reserve and economic base. Therefore, there are technological pressures between the digital economy and individual health. Differences in the educational and economic levels of the middle-aged and older groups have, to a certain extent, led to a divergence in the health effects of the digital economy. The high level of development of the digital economy is likely to create a digital divide, which will cause some middle-aged and older people to become “digital refugees.” The exclusion of this group from the digital economy and their inability to enjoy the dividends of the digital economy will have negative psychological and physical effects, thus increasing the negative impact on health. In addition, over-reliance on and use of digital technologies can similarly increase health risks for individuals (25). For example, while the digital economy provides social convenience, it crowds out the exercise time of middle-aged and older people and changes healthy lifestyles, leading to a decline in health status. Based on the above reasons, this paper proposes the following research hypotheses:

*H1*: The digital economy’s impact on health shows an inverted U-shaped relationship. Specifically, the pre-digital economy development positively affects health and the post-digital economy development negatively affects health.

### 2.2 Mechanisms of the digital economy’s impact on health

Income distribution effects. Schumpeter’s creative destruction theory believes that technological innovation is the process of destroying old structures and creating new ones. Relying on innovative technologies such as information technology, big data and cloud computing, the development of the digital economy will inevitably bring about changes in the organizational structure of production and the structure of the workforce, which is in line with the characteristics of the creative destruction theory of Schumpeter. Of these, changes in labor force structure are more strongly associated with individual health. Discussions on technological change and labor force structure have focused on substitution and creation effects (26). The substitution effect is mainly reflected in the fact that the development of technology eliminates some traditional labor jobs, especially those with weak mechanical and emotional interactions (27). Frey and Osborne utilized a Gaussian process classifier to estimate the likelihood of computerization for 702 specific occupations. Their findings revealed that approximately 47% of these occupations are projected to be automated by computers in the future (28). The creation effect is mainly manifested in the fact that technological development creates new jobs and expands the demand for employment. In the research context of this article, the impact of the digital economy on health through income can be analyzed from two perspectives, the substitution effect and the creation effect. Since the initiation of the reform and opening-up policy, the binary economic structure between rural and urban areas has long been a persistent challenge for China’s economic development. The issue of urban–rural division has resulted in a noticeable income gap between urban and rural areas. The digital economy can create more jobs by affecting market size, knowledge spillovers and factor combinations. It can also enrich employment resources through accelerated information dissemination, expanded channels, and other means. This can effectively stimulate rural residents’ willingness to start their own businesses and take up employment, which in turn raises incomes in rural areas, thus narrowing the income gap between urban and rural residents. At the same time, policies to support the development of the digital economy, such as “smart cities,” “broadband China” and other policy pilots, have increased the process of urbanization and attracted the income level of rural migrant workers who have moved to cities, which has improved the urban–rural income distribution gap to a certain extent. China’s current economic structure is still labor-intensive, but the digital economy has driven changes in production technology and accelerated the development of skill-intensive industries. In terms of the substitution effect, given that the education level of rural residents is generally lower than that of their urban counterparts, they have a limited stock of knowledge about smart technological products and services in the era of the digital economy, which makes it difficult for them to master and apply them. As a result, it is difficult for surplus rural labor to meet the urban demand for new types of talent in the digital economy. The drive for the digital economy is focused on knowledge-intensive and technology-intensive productive services and high-end services. However, rural–urban migrant workers face the problem of the digital divide, making it difficult for them to gain more opportunities in the new round of tertiary employment expansion, which brings about the so-called “reverse urbanization” problem. Thus, initial digital economic development can contribute to the urbanization process and increase the level of entrepreneurship among rural residents, thereby increasing their incomes and narrowing the income gap between urban and rural residents. However, with the further development of the digital economy, the effect of the urban–rural digital divide has become more obvious, the phenomenon of “reverse urbanization” has appeared, and the digital economic support for entrepreneurship of rural residents has gradually decreased, which has widened the income gap between urban and rural residents. Based on this, this paper puts forward the following hypotheses:

*H2*: The digital economy can affect individual health through urban–rural income distribution. Specifically, the pre-digital economy can narrow the income gap positively affecting health, and the post-digital economy can widen the income gap negatively affecting health.

Resource allocation effect. The digital economy, leveraging the advantages of internet communication and sharing, can effectively coordinate the supply and demand conflicts of healthcare facilities and improve resource allocation (1, 29, 30). Currently, the development of information technology has brought us into a new era of medical treatment, and we are constantly exploring the laws of human disease (31). The application of information technology, information related to diseases can be collected and analyzed (32). This can promote the advancement of medicine. and support the improvement of human health (33). The digital economy can help to upgrade the healthcare system and advance toward personalized and precision medicine. The digital economy is a new model that connects the virtual economy with the traditional economy, and one of its advantages is optimizing resource allocation. Through the transmission function of big data, medical services can break the limitation of diagnosis and treatment in time and area, and improve the coverage of medical services (34). Additionally, it can reduce the cost of medical services, enhance the spillover effect of high-quality medical resources, expedite the flow of medical resources to underdeveloped areas, and improve primary healthcare efficiency (35). This can improve the unequal distribution of healthcare resources and the health status of the middle-aged and older groups. Nevertheless, it is challenging to rapidly enhance the health literacy of middle-aged and older individuals through the digital economy. Spending money to cure diseases is still the way of health management for most middle-aged and older people in China, and insufficient attention is paid to disease prevention and disease monitoring. As middle-aged and older people are slow to accept new medical services and are more willing to trust physical medical institutions, they do not have a good understanding of digital healthcare. The dividend generated by the digital economy makes Internet companies turn to “Internet + healthcare,” and the pursuit of economic effects will increase the cost of healthcare, which is not conducive to the middle-aged and older groups to see the doctor and damage individual health. Based on this, this paper proposes the following hypotheses:

*H3*: The digital economy can impact individual health through healthcare resource allocation. Specifically, the pre-digital economy development positively affects the level of medical care to improve health, and the post-digital economy development negatively affects the level of medical care to the detriment of health.

### 2.3 Moderating effects of air pollution

The environment is an important factor affecting individual health. Along with the increasing environmental pollution, the impact of air pollution on individual health has received widespread attention. When discussing economic behavior and individual health, many scholars have considered environmental factors. Therefore, when discussing the relationship between the digital economy, a new economic form, and health, how air pollution affects the relationship between the two deserves further attention and consideration. This paper argues that more severe air pollution can reinforce the inverted U-shaped relationship between the digital economy and the health of middle-aged and older people. Most studies have proved that the worse the health level of people in areas with severe air pollution. In this case, the rapid development of the digital economy will improve the level of medical services and the efficiency of resource allocation, which can change people’s health level. In addition, the digital economy can also bring about environmental improvements and reduce the level of air pollution, which is also beneficial to health. Our hypothesis also assumes that when the digital economy reaches a certain stage of development, it will have a negative impact on health. Further, in the later stages of the development of the digital economy, air pollution is superimposed with the negative impacts of the digital economy, which will inevitably make the health condition worse. Based on this, this paper proposes the following hypotheses:

*H4*: Air pollution reinforces the inverted U-shaped effects of the digital economy on the health of middle-aged and older adults.

## 3 Model and data

### 3.1 Basic regression model

In order to test the nonlinear impact of the digital economy on health, we first set up a baseline OLS model for empirical analysis. Something worth noting is that we include a quadratic term for the digital economy to verify this nonlinear relationship. The inclusion of a squared term to capture the nonlinear relationship of the variables is a common practice in econometrics, and has been argued in the field of health economics based on this design (36, 37). Based on the previous theoretical model and research hypotheses, we constructed the benchmark OLS econometric model as follows:


healthit=α1+β1digitalct+γ1digitalct2+δ1Xithealthit+λ1Yct+μi+θt+πc+εit

where *i*, *t*, and *c* are categorized to denote individual, year, and time. The explanatory variable $\mathrm{healt}h_{\mathrm{it}}$ is the health level of respondent i in year t. The core explanatory variable $\mathrm{digita}l_{ct}$ denotes the level of digital economy development in city c in year t, and its quadratic term is denoted $\mathrm{digita}l_{ct}^{2}$ . $\beta_{1}$ and $\gamma_{1}$ are the coefficients that are the focus of this paper. $X_{\mathrm{it}}$ denotes an individual-level control variable. $Y_{ct}$ denotes a city-level control variable. $\mu_{i}$ , $\theta_{t}$ , and $\pi_{c}$ denote individual fixed effects, time fixed effects, and city fixed effects, respectively. $\varepsilon_{\mathrm{it}}$ is the random error term. In addition, given that the model uses panel data, in order to improve better resolution of heteroskedasticity, we use robust standard errors clustered to the individual level in all regressions.

### 3.2 Variant

Explained Variables. For the measurement of individual health, this paper uses Self-Assessed Health (SAH). Based on the CHARLS questionnaire “How do you feel about your health?,” the answers were assigned a scale of 1–5 (very bad = 1, bad = 2, fair = 3, good = 4, very good = 5). Although self-assessment of health is somewhat subjective, a large number of studies have shown that this indicator is effective in reflecting an individual’s state of health and can also provide a useful reference in predicting mortality and disease incidence (38). In addition, environmental factors are recognized as one of the most important factors affecting health. The self-assessment of health, in its characterization of health status, fits to a large extent with the need to analyze the relationship between the environment and health.

Core explanatory variables. Drawing on relevant Chinese scholars, the level of digital economy development is measured mainly in terms of the Internet and digital finance (39). The level of Internet development in cities includes four main aspects: Internet penetration, related employees, outputs and cell phone penetration rates (40). These four aspects are expressed in terms of the number of Internet broadband access subscribers per 100 people, the proportion of employees in the computer services and software industry to those in urban units, the total amount of telecommunication services *per capita*, and the number of cell phone subscribers per 100 people, respectively. Digital finance is represented by The Peking University Digital Financial Inclusion Index of China (PKU_DFIIC). The index is mainly compiled by the Digital Finance Research Center of Peking University and is widely recognized for measuring the level of digital finance development in China.

Control variables. Considering that individual health level is also affected by other factors, in order to minimize model bias, this paper includes control variables at the individual level and city level, respectively. At the individual level, age, gender (male = 1, female = 0), education level (below elementary school = 1, middle school = 2, high school and above = 3), and marital status (with spouse = 1, without spouse = 0) are controlled. At the city level, it controls for the level of economic development (GDP), population size (the number of registered persons in the city), and the level of urbanization (mainly using the number of permanent residents in towns/(the number of permanent residents in towns + the number of permanent residents in villages), and partly using the number of non-farmers/total population at the end of the year).

### 3.3 Data

The data used in this paper include both the individual micro level and the macroeconomic level. At the micro-individual level, we utilize the China Health and Retirement Longitudinal Study (CHARLS).CHARLS began in 2011, and the publicly available data have been tracked for three rounds, in 2013, 2015, and 2018, with the sample covering 25 provinces (municipalities and autonomous regions) in China. The interviews for the CHARLS response rate and data quality are among the top in similar projects in the world, and the data have been widely used and recognized in the academic community. In this paper, we organize the data from these four issues made public by CHARLS, and get a total of 54,955 observations after excluding the samples with missing variables and outliers. At the macroeconomic level, we use the community codes provided by CHARLS to get the information of the city where they are located, and then match them with the individual codes to get the final sample covering 123 cities. The data for cities are obtained from the China Urban Statistical Yearbook, EPS database, etc. The descriptive statistics of the variables are shown in Table 1.

**Table 1:** Descriptive statistics.

Variable	Obs	Mean	SD.	Min	Max
health	54,955	2.947	0.966	1	5
digital	54,955	0.117	0.085	0	0.826
Gender	54,955	0.475	0.499	0	1
edu	54,955	1.373	0.568	1	3
marital	54,955	0.812	0.39	0	1
Age	54,955	59.657	10.06	45	100
lnGDP	54,955	7.556	0.909	5.185	10.395
Population	54,955	599.534	450.769	120.1	3,404
Urbanization	54,955	0.528	0.145	0.226	1

## 4 Results of the study

### 4.1 Baseline results

Prior to regression, we tested the data for multicollinearity. Table 2 demonstrates the results of the multicollinearity, where it can be found that the VIF of all variables and their means are less than 10, indicating that there is no multicollinearity between the variables.

**Table 2:** Multicollinearity test.

Variable	VIF	1/VIF
Urbanization	2.23	0.4481
digital	2.23	0.4490
lnGDP	1.45	0.6901
Population	1.41	0.7084
Education	1.23	0.8145
Age	1.18	0.8500
Gender	1.13	0.8861
Marital	1.11	0.8988
Mean VIF	1.50	

The results of the impact of the digital economy on health are shown in Table 3. Columns (1, 2) are random effects models and column (3) is a fixed effects model. Column (1) does not include control variables and only roughly analyzes the impact of digital economy on health. The results show that the coefficient of digital is 0.3189 and significant at 1% level. The coefficient of digital_sq is −1.0578 and significant at 1% level. Column (2) further adds individual characteristics and city control variables, and the results remain similar, with coefficients of 0.5849 and − 1.1257 for digital and digital_sq, respectively, which both pass the 1% significance test. Column (3) uses a fixed effects model, specifically, the coefficient of the digital economy is significantly positive at the 1% level, and the coefficient of the quadratic term of the digital economy is significantly negative at the 1% level, suggesting that the digital economy exhibits an inverted U-shape nonlinear effect on health. H1 is confirmed. Our results provide new evidence on the health welfare effects of the digital economy. Middle-aged and older adults can positively benefit in the predevelopmental period of the digital economy. The level of development of the digital economy at this time does not have a digital divide, which can facilitate access to health advice, favor the health literacy of the older, and also support the development of digital healthcare. In contrast, in the later stages of the development of the digital economy, the health of middle-aged and older people is negatively affected. In terms of development, this is when the digital economy creates a digital divide and disadvantaged groups continue to lag behind, therefore bringing about inequalities in health literacy (41, 42).

**Table 3:** Benchmark regression results.

	(1) Health	(2) Health	(3) Health
Digital	0.3189^***^ (0.1202)	0.5849^***^ (0.1534)	1.2002^***^ (0.3935)
digital_sq	−1.0578^***^ (0.2326)	−1.1257^***^ (0.2442)	−3.1765^***^ (0.8478)
Gender		−0.1362^***^ (0.0085)	−0.1407^***^ (0.0109)
Education		−0.0320^***^ (0.0023)	−0.0280^***^ (0.0030)
Marital		0.0015 (0.0022)	−0.0008 (0.0028)
Age		0.0118^***^ (0.0004)	0.0126^***^ (0.0006)
lnGDP		−0.1570^***^ (0.0053)	−0.1477^***^ (0.0072)
Population		0.0002^***^ (0.0000)	0.0002^***^ (0.0000)
Urbanization		−0.1964^***^ (0.0446)	0.2492^*^ (0.1346)
_cons	2.9322^***^ (0.0107)	3.5305^***^ (0.0464)	3.1459^***^ (0.0999)
*N*	54,955	54,955	52,061
r2_a	0.0006	0.0500	0.0506

Standard errors in parentheses. ^*^p < 0.1, ^**^p < 0.05, ^***^p < 0.01.

### 4.2 Endogenous discussions

For the influences on an individual’s health, especially some unobservables, it is difficult to include them all in the model discussion. Therefore other key omitted variables may pose endogeneity problems. In order to mitigate the endogeneity problem as much as possible, this paper adopts the instrumental variable (IV) approach. For this purpose, we use the spherical distance between the household’s location and Hangzhou, China as an instrumental variable (43). In addition, a time-varying variable is introduced to construct the panel instrumental variable (44). Here we choose distance multiplied by the number of Internet broadband accesses to construct the instrumental variable (39). Hangzhou is China is a representative city of the development of the digital economy, the closer it is to Hangzhou, the more likely it is to be subject to spatial spillover effects, and thus have similar location advantages. The former is entirely exogenous geographic information that can measure the basis for the development of the digital economy. Digital infrastructure is an important condition for the development of the digital economy. The latter, as time-series data, can reflect the development trend of the digital economy side by side. Table 4 shows the test results of the two-stage regression method. Among them, the first stage regression results in column (1) with the digital economy as the explanatory variable and the introduction of the instrumental variable as the explanatory variable, the instrumental variable is significantly positive at the 1% level, indicating that there is a significant association between the instrumental variable and the digital economy. This is in line with our expectation that the closer the city to HCM City and the better the digital infrastructure, the better the development of the digital economy. The fitted values of digital economy are obtained and substituted into the second stage regression, and the regression results are shown in column (2). The results show that the digital economy still exhibits a significant inverted U-shaped nonlinear effect on individual health, which further strengthens the persuasiveness of the empirical results. In addition, the non-identification of instrumental variables The LM test rejected the original hypothesis, indicating that the endogenous variable of the test, digital economy, is identifiable relative to the instrumental variable; the weak instrumental variable identification Wald F-test is greater than the critical value at the given level of significance, suggesting that the instrumental variable does not have a weak instrumental variable problem in explaining the digital economy. This validates the reliability of the instrumental variable approach.

**Table 4:** Results of endogeneity discussion.

	(1) Digital	(2) Health
IV	2.21e-12^***^ (0.0000)	
Digital		8.4903^***^ (4.56)
digital_sq		−17.1903^***^ (−4.74)
Gender	−0.0000 (0.0002)	−0.1405^***^ (−12.79)
Education	−0.0000 (0.000)	−0.0278^***^ (−9.29)
Marital	−0.0000 (0.0000)	−0.0005 (−0.19)
Age	−0.0000 (0.0000)	0.0128^***^ (22.79)
lnGDP	−0.0006^***^ (0.0001)	−0.1416^***^ (−19.20)
Population	−7.20e-07^***^ (0.0000)	0.0002^***^ (16.52)
Urbanization	0.0022 (0.0018)	0.2415^*^ (1.78)
KP rk LM statistic		308.99
Cragg-Donald Wald F statistic		2203.93
*N*	52,061	52,061
r2_a	0.7828	0.0365

Standard errors in parentheses. ^*^p < 0.1, ^**^p < 0.05, ^***^p < 0.01.

### 4.3 Robustness check

In order to make the research results more stable, this paper adopts a variety of methods to carry out the robustness test in the following ways: (1) Censoring the unbalanced panel data into balanced panel data. CHARLS conducts tracking surveys in which many subjects do not appear in the follow-up surveys, while at the same time many new respondents are included. Thus CHARLS provides an unbalanced panel data set. For the robustness tests, we retained subjects in the sample that had observations in all years, resulting in a balanced panel of 14,468 observations. The regression results for the balanced panel data show that the coefficient on the digital economy is significantly positive at the 5% level and the coefficient on the digital economy quadratic term is significantly negative at the 1% level. Therefore, H1 remains valid. (2) Replacement of regression model. Since the explanatory variable individual health in this paper belongs to typical discrete variables, the ORDERED logit model can be chosen to verify the impact of the digital economy on health. The regression results based on ologit show that the difference in the measurement model does not change the baseline regression results, which indicates that the relationship between the digital economy and health analyzed in this paper is robust. (3) Adding new control variables. Based on the benchmark model, this paper adds new control variables at the individual level and city level to examine the robustness of the regression results. Specifically, this paper adds household consumption level (ctot) at the individual level and wage level (wage) at the city level. After adding the control variables separately, the coefficients of the digital economy are all significantly positive at the 1% level, and the coefficients of the quadratic terms of the digital economy are all significantly negative at the 1% level. The results are consistent with the above, further providing conviction to the results (Table 5).

**Table 5:** Robustness test results.

	(1) Health	(2) Health	(3) Health	(4) Health
Digital	2.0043^**^ (0.7837)	2.2580^***^ (0.7648)	1.7028^***^ (0.4938)	1.1714^***^ (2.97)
digital_sq	−4.6981^***^ (1.6833)	−5.6282^***^ (1.5976)	−3.8922^***^ (1.0599)	−3.1432^***^ (−3.70)
Gender	−0.1368^***^ (0.0191)	−0.2829^***^ (0.0228)	−0.1471^***^ (0.0135)	−0.1421^***^ (−12.84)
Education	−0.0325^***^ (0.0052)	−0.0585^***^ (0.0061)	−0.0274^***^ (0.0037)	−0.0276^***^ (−9.09)
Marital	−0.0005 (0.0049)	−0.0010 (0.0059)	−0.0005 (0.0037)	−0.0009 (−0.31)
Age	0.0107^***^ (0.0010)	0.0255^***^ (0.0012)	0.0129^***^ (0.0007)	0.0129^***^ (22.95)
lnGDP	−0.1119^***^ (0.0128)	−0.2949^***^ (0.0150)	−0.1567^***^ (0.0092)	−0.1416^***^ (−16.72)
Population	0.0002^***^ (0.0000)	0.0004^***^ (0.0000)	0.0002^***^ (0.0000)	0.0002^***^ (15.49)
Urbanization	−0.1071 (0.2398)	0.5721^**^ (0.2825)	0.0190 (0.1714)	0.2526^*^ (1.86)
ctot			−0.0000 (0.0000)	
wage				−0.0000^*^ (−1.69)
_cons	3.1428^***^ (0.1758)		3.2618^***^ (0.1270)	3.1149^***^ (0.1018)

Standard errors in parentheses. ^*^p < 0.1, ^**^p < 0.05, ^***^p < 0.01.

### 4.4 Further analysis from an air pollution perspective

As mentioned earlier, the negative health effects of air pollution are well known. Next, this paper further analyzes the health effects of the digital economy from an air pollution perspective. For the measurement of air pollution, we use the concentration level of urban pollutant PM2.5 as a proxy. The PM2.5 pollution data in this paper comes from a rasterized dataset of global historical annual average PM2.5 published by the Center for International Earth Science Information Network (CIESIN) at Columbia University. It should be noted that, because of the inverted U-shaped relationship between the digital economy and individual health, this paper is divided into constructing the interaction term between the digital economy and its secondary term with health, and by testing the relationship between the interaction term and health, as a way to explore the moderating effect of air pollution on the inverted U-shaped relationship between the digital economy and health. The regression results are shown in Table 6.

**Table 6:** Results of moderating effects analysis.

	(1) Health	(2) Health
Pollution	−0.0012^***^ (0.0003)	−0.0011^***^ (0.0003)
Digital		1.2744^***^ (0.4110)
digital_sq		−3.3133^***^ (0.9179)
dig_po		0.0223^**^ (0.0102)
digsq_po		−0.0380^**^ (0.0187)
Gender	−0.1400^***^ (0.0109)	−0.1401^***^ (0.0109)
Education	−0.0283^***^ (0.0030)	−0.0282^***^ (0.0030)
Marital	−0.0008 (0.0028)	−0.0007 (0.0028)
Age	0.0125^***^ (0.0006)	0.0125^***^ (0.0006)
lnGDP	−0.1449^***^ (0.0072)	−0.1448^***^ (0.0073)
Population	0.0002^***^ (0.0000)	0.0002^***^ (0.0000)
Urbanization	0.2687^**^ (0.1338)	0.2783^**^ (0.1354)
_cons	3.2510^***^ (0.0930)	3.1568^***^ (0.1018)
*N*	52,061	52,061
r2_a	0.0506	0.0510

Standard errors in parentheses. ^*^p < 0.1, ^**^p < 0.05, ^***^p < 0.01.

In column (1) we verify the effect of air pollution on health. The regression coefficient for air pollution is −0.0012 and passes the significance test at 1% level. This indicates that air pollution presents a significant negative effect on individual health, which is consistent with existing studies. Column (2) incorporates the interaction term between air pollution and digital economy, which verifies the moderating effect of air pollution on digital economy and health. Specifically, the coefficient of the interaction term between digital economy and air pollution is 0.0223 and significant at the 5% level. This indicates that the interaction between digital economy and air pollution has a significant positive effect on health, i.e., air pollution positively reinforces the relationship between digital economy and health. The coefficient of the interaction term between the quadratic term of digital economy and air pollution is −0.038 and significant at 5% level. This indicates that after a certain level of development of digital economy, air pollution positively moderates the relationship between digital economy and health, i.e., it strengthens the negative relationship between digital economy and health.

### 4.5 Mechanism analysis

Based on the theoretical mechanism analysis and benchmark regression results above, we find that the digital economy has a significant inverted U-shaped nonlinear effect on individual health, which is a new finding different from the existing studies. We believe that the mechanism leading to this inverted U-shaped nonlinear effect may be caused by income distribution and health care resources. In the early stage of the development of the digital economy, the digital economy improves individual health by narrowing the income gap between urban and rural areas and increasing the level of healthcare. However, as the digital economy develops, the digital divide widens, which leads to a negative impact on individual health by widening the urban–rural income gap and unfavorable allocation of healthcare.

Column (1) shows income inequality as a mechanism. Three indicators in the existing literature to measure the urban–rural income gap are the ratio of disposable income *per capita* in urban areas to disposable income *per capita* in rural areas, the Gini coefficient and the Thiel index. The ratio of disposable income *per capita* in urban and rural areas is a static indicator that does not take into account the differences in population shares between urban and rural areas, and therefore does not reflect the mobility of the population between urban and rural areas. The Gini coefficient is mainly used to measure the overall income gap and is more sensitive to income changes in the middle class, while the urban–rural income gap mainly exists at the two ends of the spectrum, so the Gini coefficient cannot fully reflect the urban–rural income gap. In contrast, the Tyrell index is more sensitive to the income changes of high-income and low-income classes, so this paper chooses the Tyrell index to measure the urban–rural income gap in China. The Thiel index is a positive indicator, the larger the value, the larger the urban–rural income gap. Its calculation formula is:


$$\mathrm{Thei}l_{\mathrm{it}}=\overset{2}{\underset{i=1}{\sum }}\left(\frac{y_{\mathrm{it}}}{y_{t}}\right)\times \ln\left[\frac{y_{\mathrm{it}}}{y_{t}}/\frac{x_{\mathrm{it}}}{x_{t}}\right]$$

where *i* = 1 and *i* = 2 denote urban and rural areas, respectively, t denotes year, y denotes disposable income, and x denotes population size.

From the results, the coefficient of digital economy is −0.1039 and significantly positive at 1% level. This indicates that the development of digital economy can curb urban–rural income inequality, and when income distribution inequality is alleviated, it thus has a positive impact on health. The coefficient of the quadratic term of digital economy is 0.2617 and is significantly positive at 1% level. This suggests that the later stages of the development of the digital economy can widen income inequality to the extent that it has a negative impact on health. This suggests that urban–rural income disparity is the pathway through which the digital economy affects health.

Column (2) shows the mechanism of medical resources. Restricted to the limited data on healthcare resources for prefecture-level cities in the China Urban Statistical Yearbook, we use data on the number of doctors and the number of beds to characterize basic healthcare resources. Specifically, the two indicators, the number of beds in healthcare institutions and the number of doctors in healthcare institutions, are dimensionless, and the average variance contribution rate is used as the weight to calculate the composite value of the level of supply of basic healthcare resources in each region, in order to reflect the level of basic healthcare resources in each region.

From the results, the coefficient of digital economy is 0.5080 and is significantly positive at 1% level. This indicates that the development of digital economy improves the healthcare resources and when the healthcare resources are improved, thus it has a positive impact on health. The coefficient of the quadratic term of digital economy is −0.2527 and is significantly positive at 1% level. This indicates that the later stages of the digital economy development will have a negative impact on healthcare resources, which in turn will have a negative impact on health. This suggests that healthcare resources are the path of action of the digital economy affecting health (Table 7).

**Table 7:** Results of mechanism of action analysis.

	(1) Equality	(2) Medical
Digital	−0.1039^***^ (0.0059)	0.5080^***^ (0.0485)
digital_sq	0.2617^***^ (0.0160)	−0.2527^***^ (0.0821)
Gender	−0.0001 (0.0001)	−0.0052^***^ (0.0019)
Education	0.0002^***^ (0.0000)	0.0026^***^ (0.0005)
Marital	0.0000 (0.0000)	−0.0003 (0.0004)
Age	−0.0000^**^ (0.0000)	0.0000 (0.0001)
lnGDP	0.0007^***^ (0.0001)	0.0060^***^ (0.0011)
Population	−0.0000^***^ (0.0000)	0.0000 (0.0000)
Urbanization	0.0011 (0.0022)	0.0922^***^ (0.0250)
_cons	0.1008^***^ (0.0013)	9.0862^***^ (0.0138)
*N*	52,061	52,061
r2_a	0.9626	0.9714

Standard errors in parentheses. ^*^p < 0.1, ^**^p < 0.05, ^***^p < 0.01.

### 4.6 Heterogeneity analysis

In order to investigate whether there are differences in the impact of the digital economy on the health of individuals, this paper discusses four aspects of gender, education level, place of residence, and geographic location of the city, and the specific heterogeneity is analyzed as follows:

Columns (1) and (2) of Table 8 show the gender differences in the impact of the digital economy on health. For females, the coefficient of digital economy is 2.9428 and significant at 1% level and the coefficient of digital economy quadratic term is −6.3216 and significant at 1% level. For males, the coefficient of digital economy is 1.1342, and the coefficient of quadratic term of digital economy is −2.4354 and significant at 1% level. This indicates that the relationship between digital economy and health is stronger in the female group. Columns of table (3–5) show the differences in the impact of digital economy on health across different levels of education. For people with low education level, the effect of digital economy on health is not significant, and the coefficient of the quadratic term of digital economy is significantly negative at 5% level, which indicates that the development of digital economy does not have any effect on individual’s health at the initial stage, and it will have an inhibitory effect on individual’s health after a certain level of development. For people with secondary education level, digital economy is not significantly associated with individual health. For people with higher education level, the coefficient of digital economy is significantly negative at 5% level and the coefficient of digital economy quadratic term is significantly positive at 10% level. The comparison shows that the impact of the digital economy on the higher education level group is significantly different from that of the lower and middle education level groups.

**Table 8:** Heterogeneity analysis based on gender and education level.

	Sex		Education level		
	Female	Male	Low	Middle	High
	(1)	(2)	(3)	(4)	(5)
Digital	2.9428^***^ (0.6714)	1.1342 (0.7400)	0.6423 (0.5427)	−0.9487 (1.4326)	−25.9688^**^ (12.1538)
digital_sq	−6.3216^***^ (1.5297)	−2.4354^***^ (1.5503)	−2.2809^*^ (1.1918)	1.2241 (3.1113)	50.8785^*^ (26.7211)
Gender			−0.1245^***^ (0.0151)	−0.1869^***^ (0.0316)	−0.1576 (0.2556)
Education	−0.0242^***^ (0.0050)	−0.0332^***^ (0.0058)			
Marital	−0.0037 (0.0044)	0.0050 (0.0059)	−0.0010 (0.0036)	0.0045 (0.0113)	−0.0920 (0.0796)
Age	0.0115^***^ (0.0010)	0.0149^***^ (0.0010)	0.0132^***^ (0.0007)	0.0153^***^ (0.0019)	0.0251^***^ (0.0083)
lnGDP	−0.1639^***^ (0.0123)	−0.1268^***^ (0.0134)	−0.1876^***^ (0.0101)	−0.0974^***^ (0.0218)	0.0724 (0.1071)
Population	0.0002^***^ (0.0000)	0.0002^***^ (0.0000)	0.0002^***^ (0.0000)	0.0002^***^ (0.0000)	0.0001 (0.0002)
Urbanization	0.2434 (0.2171)	0.0185 (0.2449)	0.3358^*^ (0.2009)	0.4259 (0.4020)	0.1722 (1.1306)
_cons	3.1898^***^ (0.1643)	2.8305^***^ (0.1879)	3.3280^***^ (0.1438)	2.5517^***^ (0.3043)	2.5748 (1.5540)
*N*	20,264	17,191	29,914	6,929	225
r2_a	0.0308	0.0425	0.0380	0.0114	−0.4810

Standard errors in parentheses. ^*^p < 0.1, ^**^p < 0.05, ^***^p < 0.01.

Table 9 show how the impact of the digital economy on health varies by place of residence and urban geographic location. For those in rural areas, the coefficient on the digital economy is 0.8343, and the coefficient on the quadratic term of the digital economy is −1.6821 and significant at the 10% level. For people in urban areas, the coefficient of digital economy is 1.4281 and significant at 1% level and the coefficient of quadratic term of digital economy is −3.8253 and significant at 1% level. This suggests that the relationship between the digital economy and health is stronger in urban areas. In the light of current developments, policy pilots to promote the development of the digital economy have focused mainly on urban areas, such as the “broadband China” pilot and the “smart city” pilot. Therefore, the link between the digital economy and individual health is closer in urban areas. In addition, we analyze the regional heterogeneity of the impact of the digital economy on health by dividing the investigators’ cities into three ranges: east, middle and west. From the coefficient of the digital economy, the regression results of the subgroups in the western region are significantly positive at the 5% level, which indicates that in the early stage of the development of the digital economy, the digital economy has a significant positive impact on the level of individual health in the western region, and does not have a significant impact on the eastern and central regions. From the coefficient of the quadratic term of the digital economy, the regression results of the subgroups in the eastern region are significantly negative at the 5% level, which indicates that in the late stage of the development of the digital economy, the digital economy has a significant negative impact on the level of individual health in the eastern region, and has no significant impact on the central and western regions. From the viewpoint of the early stage of digital economy development, the western region is more remote, the level of urbanization and medical conditions are relatively poor, the dividends of the digital economy can be fully released, and there is a significant impact on the level of individual health. From the late stage of digital economy development, the eastern region has a good economic foundation, and the digital economy can significantly widen the digital divide, so it has a significant negative impact on individual health.

**Table 9:** Heterogeneity analysis based on residence and urban location.

	Residence		Region		
	Rural	Town	East	Central	West
	(1)	(2)	(3)	(4)	(5)
Digital	0.8343 (1.8567)	1.4281^***^ (0.4721)	0.5350 (0.5213)	−2.5443 (1.6538)	2.3998^**^ (1.0284)
digital_sq	−1.6821^*^ (3.5370)	−3.8253^***^ (1.0198)	−2.1549^**^ (1.0037)	7.4950 (4.9584)	−4.3255 (3.1208)
Gender	−0.1355^***^ (0.0409)	−0.1584^***^ (0.0145)	−0.1610^***^ (0.0173)	−0.1200^***^ (0.0190)	−0.1393^***^ (0.0209)
Education	−0.0186^*^ (0.0111)	−0.0191^***^ (0.0045)	−0.0214^***^ (0.0049)	−0.0255^***^ (0.0051)	−0.0409^***^ (0.0057)
Marital	−0.0272^**^ (0.0119)	−0.0011 (0.0036)	0.0031 (0.0042)	0.0011 (0.0050)	−0.0108^*^ (0.0056)
Age	0.0169^***^ (0.0021)	0.0130^***^ (0.0008)	0.0127^***^ (0.0009)	0.0114^***^ (0.0010)	0.0138^***^ (0.0011)
lnGDP	−0.0485^*^ (0.0293)	−0.1642^***^ (0.0097)	−0.1326^***^ (0.0105)	−0.1115^***^ (0.0160)	−0.1811^***^ (0.0146)
Population	0.0002^***^ (0.0000)	0.0002^***^ (0.0000)	0.0002^***^ (0.0000)	0.0001^***^ (0.0000)	0.0003^***^ (0.0000)
Urbanization	0.3325 (0.6540)	0.0072 (0.1809)	0.3192 (0.2218)	0.7896^***^ (0.2496)	−0.2241 (0.2343)
_cons	2.0240^***^ (0.4538)	3.3581^***^ (0.1332)	2.9741^***^ (0.1671)	2.9677^***^ (0.2254)	3.4781^***^ (0.1644)
*N*	4,061	33,574	21,430	16,940	13,691
r2_a	0.4910	0.4407	0.4000	0.3750	0.3974

Standard errors in parentheses. ^*^p < 0.1, ^**^p < 0.05, ^***^p < 0.01.

## 5 Expansive analysis: the impact of the digital economy on physical health

In the above section, we have focused on characterizing the health of middle-aged and older adults through self-assessed health. While self-assessed health is appropriate as a measure of health, however, some studies have shown that self-assessed health and physical health do differ somewhat in health assessment. Considering physical health as an important aspect of health, we therefore made a brief extended analysis in this section, focusing on examining the impact of the digital economy on physical health. Following the previous measures, the following regression model was designed to examine the relationship between the two:


dmit=α2+β2digitalct+γ2digitalct2+δ2Xitdmit+λ2Yct+μi+θt+πc+εit

where $dm_{\mathrm{it}}$ denotes the individual’s physiological health level, and the rest as above. Regarding the measure of physiological health, we calculated it using the Mahalanobis distance (DM) method proposed by Cohen et al. (45). Previous studies have confirmed that this method is well represented in predicting physiological health or measuring human aging (46–48). Specifically, by calculating the distance of a biomarker from a spatial ideal health point. A larger distance indicates a poorer state of physical health. The specific calculation formula is as follows:


$$D_{M}\left(x\right)=\sqrt{{\left(x-\mu \;\right)}^{T}S^{-1}\left(x-\mu \right)}$$

where x is a vector of biomarker values for a given participant, is an equal-length vector of headcount averages for each variable representing health level, and *S* is the headcount variance–covariance matrix for the variable. We chose data from 15 biomarker records in the CHARLS database to calculate physiologic disorders. Also, to ensure the robustness of the results, we excluded the 15 biomarkers with more missing values for secondary validation.

The regression results are shown in Table 10. In column (1), the coefficient of the primary term of the digital economy is 5.3369 and significant at the 5% level, and the coefficient of the secondary term of the digital economy is insignificant. In column (2), the coefficient of the primary term of the digital economy is 5.3252 and significant at the 10% level, while the coefficient of the secondary term of the digital economy is insignificant. This indicates that the development of digital economy is linear for physical health and the level of development of digital economy negatively affects physical health. This suggests that there is a difference in the development of the digital economy on the multidimensional health of middle-aged and older people, which is manifested in the fact that it is non-linear for self-assessed health and linear for physiological health. From the current research, few studies have discussed the impact of the digital economy on health and have not provided evidence of the existence of divergence. Our extended analysis has implications for rationalizing the digital economy and focusing on health inequalities.

**Table 10:** Impact of the digital economy on physical health.

	(1) dm_1	(2) dm_2
Digital	5.3369^**^ (2.6130)	5.3252^*^ (3.0984)
digital_sq	−8.9547 (6.1307)	−7.2515 (7.1075)
Gender	0.0341 (0.0450)	0.0289 (0.0562)
Education	−0.0189 (0.0127)	−0.0049 (0.0155)
Marital	−0.0112 (0.0109)	−0.0061 (0.0132)
Age	−0.0021 (0.0022)	0.0019 (0.0026)
lnGDP	0.0441 (0.0389)	0.0172 (0.0486)
Population	0.0002 (0.0001)	0.0001 (0.0001)
Urbanization	0.6510 (0.4136)	0.9599^*^ (0.5340)
_cons	−0.9830^**^ (0.4243)	−1.2485^**^ (0.5453)
*N*	4,340	2,816
r2_a	−0.0293	−0.0373

Standard errors in parentheses. ^*^p < 0.1, ^**^p < 0.05, ^***^p < 0.01.

## 6 Conclusions and implications

Based on Chinese city-level macro data and CHARLS micro data, this paper combines the digital economy and individual health, and explores the impact of digital economic development on individual health and its internal mechanism. And based on the perspective of air pollution, the moderating effect between the two is investigated. The results found that (1) the development of China’s digital economy is an important factor affecting individual health, but it is not the higher level of the digital economy that improves individual health, and the impact of the digital economy on individual health shows a significant inverted U-shaped nonlinear characteristic. (2) In terms of the influence mechanism, the urban–rural income gap and basic medical resources are the mechanisms that lead to the inverted U-shaped influence of the digital economy on individual health. In the early stage of the development of digital economy, it will promote the improvement of individual health by improving the urban–rural income gap and basic medical resources, and in the late stage of the development of digital economy, it will negatively affect individual health by expanding the urban–rural income gap and suppressing basic medical resources. (3) Air pollution positively moderates the impact of the digital economy on health, reinforcing the inverted U-shaped relationship between the two. (4) Regarding the heterogeneity discussion, the relationship between digital economy and health is closer in the female group. The impact is significantly different for groups with higher education levels than for groups with lower and middle education levels. The relationship between the digital economy and health is stronger in urban areas. In the early stage of the development of the digital economy, the digital economy has a significant positive impact on the health of individuals in the western region, and has no significant impact on the eastern and central regions. In the late stage of the development of the digital economy, the digital economy has a significant negative effect on the level of individual health in the eastern region, and has no significant effect on the central and western regions. (5) Expansive analyses indicate that the digital economy has a negative impact on physiological health.

Based on the above conclusions, we get the following insights:

First, the Chinese government should correctly recognize the double-edged sword effect of the digital economy and rationally lay out the development path of the digital economy. Based on the actual development of the digital economy, it should optimize the allocation mechanism of regional income and medical resources. Although the development of the digital economy is beneficial to the economy and society, this paper finds that the development of the digital economy has a negative impact on the health of the middle-aged and the older in the late stage of development. If the current allocation mechanism of income and medical resources is not improved, the digital economy will harm the health of the population in the long-term development. Therefore, it is important to give full play to the advantages of the digital economy and minimize the risks and challenges it may bring.

Second, the government needs to increase investment in digital infrastructure development in rural and less developed areas to reduce the urban–rural income gap and lay a good foundation for realizing the inclusive development of the digital economy. The research in this paper finds that the digital economy can affect health by improving the income gap and improving the allocation of medical resources. Therefore, it is necessary to further improve the social security system, improve the medical resource allocation system, and improve the level of medical protection for residents to ensure that the development of the digital economy will not exacerbate the gap between the rich and the poor and the imbalance of medical resources in the long-term development.

Third, the Chinese government needs to pay attention to the heterogeneity of the role of the digital economy and rationalize its development. The research in this paper finds that there is heterogeneity in the relationship between the digital economy and the health of middle-aged and older people. It can be started from two aspects. One is to further improve the training and education system of digital technology. Focus on providing popularized education and training in digital technology for people with low education level, so that the low education level group can better adapt to the development of digital economy and narrow the digital divide. For the high-level education group, the government should encourage higher education institutions to establish cooperative relationships with digital economy enterprises, promote the transfer and application of knowledge and technology, and cultivate the innovation ability and entrepreneurship of the higher education level group, so as to further promote the high-quality development of the digital economy. Secondly, relevant digital health management platforms and applications should be further promoted to encourage residents to actively participate in self-health management and to improve self-managed health awareness and preventive healthcare capabilities.

## Data availability statement

Publicly available datasets were analyzed in this study. This data can be found here: http://charls.pku.edu.cn/.

## Author contributions

JW: Writing – original draft. QL: Writing – review & editing.

## References

[1] Luo K Liu YB Chen PF Zeng ML. Assessing the impact of digital economy on green development efficiency in the Yangtze River Economic Belt. Energy Econ. (2022) 112:106127. doi: 10.1016/j.eneco.2022.106127

[2] Tapscott D. The digital economy: promise and peril in the age of networked intelligence. Choice Rev. (1996) 33:5199. doi: 10.5860/choice.33-5199

[3] OECD. Measuring the digital economy: A new perspective. Paris: OECD Publishing (2014).

[4] Autor D Dorn D Hanson G. When work disappears: manufacturing decline and the falling marriage market value of young men. Am Econ Rev. (2019) 1:161–78. doi: 10.1257/aeri.20180010

[5] Colantone I Crinò R Ogliari L. Globalization and mental distress. J Int Econ. (2019) 119:181–207. doi: 10.1016/j.jinteco.2019.04.008

[6] McManus TC Schaur G. The effects of import competition on worker health. J Int Econ. (2016) 102:160–72. doi: 10.1016/j.jinteco.2016.06.003 30230125

[7] Coneus K Spiess CK. Pollution exposure and child health: evidence for infants and toddlers in Germany. J Health Econ. (2012) 31:180–96. doi: 10.1016/j.jhealeco.2011.09.006, PMID: 22030091

[8] Afroz R Hassan MN Ibrahim NA. Review of air pollution and health impacts in Malaysia. Environ Res. (2003) 92:71–7. doi: 10.1016/S0013-9351(02)00059-2 12854685

[9] Brunekreef B Holgate ST. Air pollution and health. Lancet. (2002) 360:1233–42. doi: 10.1016/S0140-6736(02)11274-8 12401268

[10] FullerRLandriganPJBalakrishnanKBathanGBose-O'ReillySBrauerM. Pollution and health: a progress update. Lancet Planetary health. (2022) 6:e535–47. doi: 10.1016/S2542-5196(22)00090-0, PMID: 35594895

[11] Landrigan PJ Fuller R. Pollution, health and development: the need for a new paradigm. Rev Environ Health. (2016) 31:121–4. doi: 10.1515/reveh-2015-0070 26943599

[12] Mortimer K Gordon SB Jindal SK Accinelli RA Balmes J Martin WJ. Household Air Pollution Is a Major Avoidable Risk Factor for Cardiorespiratory Disease. Chest. 142, 1308–1315. doi: 10.1378/chest.12-1596, PMID: 23131939 PMC5991547

[13] Arora S. Health, human productivity, and long-term economic growth. J Econ Hist. (2001) 61:699–749. doi: 10.1017/S0022050701030054

[14] Wu L Wan X Jahanger A Li M Murshed M Balsalobre-Lorente D. Does the digital economy reduce air pollution in China? A perspective from industrial agglomeration. Energy Rep. (2023) 9:3625–41. doi: 10.1016/j.egyr.2023.02.031

[15] Zhang Y Ran C. Effect of digital economy on air pollution in China? New evidence from the “National big Data Comprehensive Pilot Area” policy. Econ Anal Policy. (2023) 79:986–1004. doi: 10.1016/j.eap.2023.07.007

[16] Carstensen LL Fung HH Charles ST. Socioemotional selectivity theory and the regulation of emotion in the second half of life. Motiv Emot. (2003) 27:103–23. doi: 10.1023/A:1024569803230

[17] Caprara GV Steca P Alessandri G Abela JR McWhinnie CM. Positive orientation: explorations on what is common to life satisfaction, self-esteem, and optimism. Epidemiologia E Psichiatria Sociale. (2010) 19:63–71. doi: 10.1017/S1121189X00001615, PMID: 20486425

[18] Khvorostianov N. “Thanks to the internet, we remain a family”: ICT domestication by elderly immigrants and their families in Israel. J Fam Commun. (2016) 16:355–68. doi: 10.1080/15267431.2016.1211131

[19] Fang Y Chau AKC Wong A Fung HH Woo J. Information and communicative technology use enhances psychological well-being of older adults: the roles of age, social connectedness, and frailty status. Aging Ment Health. (2018) 22:1516–24. doi: 10.1080/13607863.2017.1358354, PMID: 28777010

[20] Nimrod G. Aging well in the digital age: Technology in Processes of selective optimization with compensation. J Gerontol Series B Psychol Sci Soc Sci. (2020) 75:2008–17. doi: 10.1093/geronb/gbz111, PMID: 31504873

[21] Wamsley D Chin-Yee B. COVID-19, digital health technology and the politics of the unprecedented. Big Data Soc. (2021) 8:205395172110194. doi: 10.1177/20539517211019441

[22] Frank SR. Digital health care--the convergence of health care and the internet. J Ambul Care Manage. (2000) 23:8–17. doi: 10.1097/00004479-200004000-00003 10848396

[23] Whitelaw S Mamas MA Topol E Van Spall HGC. Applications of digital technology in COVID-19 pandemic planning and response. Lancet Digital Health. (2020) 2:E435–40. doi: 10.1016/S2589-7500(20)30142-4 32835201 PMC7324092

[24] He Y Li K Wang YP. Crossing the digital divide: the impact of the digital economy on elderly individuals? Consumption upgrade in China. Technol Soc. (2022) 71:102141. doi: 10.1016/j.techsoc.2022.102141

[25] Allcott H Braghieri L Eichmeyer S Gentzkow M. The welfare effects of social media. Am Econ Rev. (2020) 110:629–76. doi: 10.1257/aer.20190658

[26] Carneiro P Lee S. Trends in quality-adjusted skill Premia in the United States, 1960-2000. Am Econ Rev. (2011) 101:2309–49. doi: 10.1257/aer.101.6.2309

[27] Acemoglu D Restrepo P. The race between man and machine: implications of technology for growth, factor shares, and employment. Am Econ Rev. (2018) 108:1488–542. doi: 10.1257/aer.20160696

[28] Frey CB Osborne MA. The future of employment: how susceptible are jobs to computerisation? Technol Forecast Soc Chang. (2017) 23:8–17.

[29] Luo AJ Qin L Yuan YF Yang ZZJ Liu F Huang PH. The effect of online health information seeking on physician-patient relationships: systematic review. J Med Internet Res. (2022) 24:e23354. doi: 10.2196/23354, PMID: 35142620 PMC8874798

[30] Wu HT Ba N Ren SY Xu L Chai JX Irfan M. The impact of internet development on the health of Chinese residents: transmission mechanisms and empirical tests. Socio Econ Plan Sci. (2022) 81:101178. doi: 10.1016/j.seps.2021.101178

[31] Li P Luo Y Yu X Wen J Jalali MS. Patients' perceptions of barriers and facilitators to the adoption of E-hospitals: cross-sectional study in Western China. J Med Internet Res. (2020) 22:e17221. doi: 10.2196/17221, PMID: 32525483 PMC7317627

[32] Zillien N Hargittai E. Digital distinction: status-specific types of internet usage. Soc Sci Q. (2009) 90:274–91. doi: 10.1111/j.1540-6237.2009.00617.x

[33] Nevado-Pena D Lopez-Ruiz VR Alfaro-Navarro JL. Improving quality of life perception with ICT use and technological capacity in Europe. Technol Forecast Soc Chang. (2019) 148:119734–119734.119711. doi: 10.1016/j.techfore.2019.119734

[34] Choi N. Relationship between health service use and health information technology use among older adults: analysis of the US National Health Interview Survey. J Med Internet Res. (2011) 13:e33. doi: 10.2196/jmir.1753, PMID: 21752784 PMC3221375

[35] Andersson G Titov N Dear BF Rozental A Carlbring P. Internet-delivered psychological treatments: from innovation to implementation. World Psychiatry. (2019) 18:20–8. doi: 10.1002/wps.20610, PMID: 30600624 PMC6313242

[36] Liu B Chen H Wang Y. More work, better health? The moderation effect of employee-organizational psychological distance. J Health Psychol. (2020) 26:2200–12. doi: 10.1177/1359105320906244, PMID: 32122173

[37] Siciliani L Stanciole A Jacobs R. Do waiting times reduce hospital costs? J Health Econ. (2009) 28:771–80. doi: 10.1016/j.jhealeco.2009.04.002, PMID: 19446901

[38] Mossey JM Shapiro E. Self-rated health: a predictor of mortality among the elderly. Am J Public Health. (1982) 72:800–8. doi: 10.2105/AJPH.72.8.800, PMID: 7091475 PMC1650365

[39] Tao Z Zhi Z Shangkun L. Digital economy, entrepreneurship, and high-quality economic development: empirical evidence from urban China. Manage World. (2020) 36:65–76. doi: 10.19744/j.cnki.11-1235/f.2020.0154

[40] Qunhui H Yongze Y Songlin Z. Internet development and productivity growth in manufacturing industry: internal mechanism and China experiences. China Indust Econ. (2019) 8:5–23. doi: 10.19581/j.cnki.ciejournal.2019.08.001

[41] Chesser A Burke A Reyes J Rohrberg T. Navigating the digital divide: a systematic review of eHealth literacy in underserved populations in the United States. Informat Health Social Care. (2015) 41:1–19. doi: 10.3109/17538157.2014.948171, PMID: 25710808

[42] Levy H Janke AT Langa KM. Health literacy and the digital divide among older Americans. J Gen Intern Med. (2014) 30:284–9. doi: 10.1007/s11606-014-3069-5, PMID: 25387437 PMC4351282

[43] Xun Z Guanghua W Jiajia Z Zongyue H. Digital economy, financial inclusion, and inclusive growth. Econ Res J. (2019) 54:71–86. doi: 10.19602/j.chinaeconomist.2020.05.07

[44] Nunn N Qian N. US food aid and civil conflict. Am Econ Rev. (2014) 104:1630–66. doi: 10.1257/aer.104.6.1630

[45] Cohen AA Milot E Yong J Seplaki CL Fulop T Bandeen-Roche K. A novel statistical approach shows evidence for multi-system physiological dysregulation during aging. Mech Ageing Dev. (2013) 134:110–7. doi: 10.1016/j.mad.2013.01.004 23376244 PMC3971434

[46] Li Q Legault V Girard VD Ferrucci L Fried LP Cohen AA. An objective metric of individual health and aging for population surveys. Popul Health Metrics. (2022) 20:289. doi: 10.1186/s12963-022-00289-0 PMC8974028 35361249

[47] Li Q Legault V Honfo SH Milot E Jia Q Wang F. Physiological dysregulation proceeds and predicts health outcomes similarly in Chinese and Western populations. J Gerontol A Biol Sci Med Sci. (2023):glad146. doi: 10.1093/gerona/glad146, PMID: 37313838 PMC11491748

[48] Li Q Wang SR Milot E Bergeron P Ferrucci L Fried LP. Homeostatic dysregulation proceeds in parallel in multiple physiological systems. Aging Cell. (2015) 14:1103–12. doi: 10.1111/acel.12402, PMID: 26416593 PMC4693454

